# Exposure to Sunlight Reduces the Risk of Myopia in *Rhesus Monkeys*

**DOI:** 10.1371/journal.pone.0127863

**Published:** 2015-06-01

**Authors:** Yong Wang, Hui Ding, William K. Stell, Liangping Liu, Saiqun Li, Hongshan Liu, Xingwu Zhong

**Affiliations:** 1 Zhongshan Ophthalmic Center and State Key Laboratory of Ophthalmology, Sun Yat-sen University, Guangzhou, China; 2 Hainan Eye Hospital, Zhongshan Ophthalmic Center, Sun Yat-sen University, Haikou, Hainan Province, China; 3 Department of Cell Biology and Anatomy, University of Calgary—Cumming School of Medicine, Calgary, Alberta, Canada; 4 Department of Surgery, University of Calgary—Cumming School of Medicine, Calgary, Alberta, Canada; Wenzhou Medical University, CHINA

## Abstract

Exposure to sunlight has recently been postulated as responsible for the effect that more time spent outdoors protects children from myopia, while early life exposure to natural light was reported to be possibly related to onset of myopia during childhood. In this study, we had two aims: to determine whether increasing natural light exposure has a protective effect on hyperopic defocus-induced myopia, and to observe whether early postnatal exposure to natural light causes increased risk of refractive error in adolescence. Eight rhesus monkeys (aged 20-30 days) were treated monocularly with hyperopic-defocus (-3.0D lens) and divided randomly into two groups: AL group (n=4), reared under Artificial (indoor) Lighting (08:00-20:00); and NL group (n=4), exposed to Natural (outdoor) Light for 3 hours per day (11:00-14:00), and to indoor lighting for the rest of the light phase. After being reared with lenses for ca. 190 days, all monkeys were returned to unrestricted vision until the age of 3 years. Another eight age-matched monkeys, reared with unrestricted vision under artificial lighting since birth, were employed as controls. The ocular refraction, corneal curvature and axial dimensions were measured before lens-wearing (at 23±3 days of age), monthly during the light phase, and at the age of puberty (at 1185+3 days of age). During the lens-wearing treatment, infant monkeys in the NL group were more hyperopic than those in the AL group (F=5.726, *P*=0.032). Furthermore, the two eyes of most NL monkeys remained isometropic, whereas 3 of 4 AL monkeys developed myopic anisometropia more than -2.0D. At adolescence, eyes of AL monkeys showed significant myopic anisometropia compared with eyes of NL monkeys (AL vs NL: -1.66±0.87D vs -0.22±0.44D; *P*=0.002) and controls (AL vs Control: -1.66±0.87D vs -0.05±0.85D; *P*<0.0001). All differences in refraction were associated with parallel changes in axial dimensions. Our results suggest that exposure to natural outdoor light might have an effect to reduced hyperopic defocus-induced myopia. Also, the data imply that early life exposure to sunlight may help to maintain normal development of emmetropization later in life, and thus lower the risk of myopic anisometropia in adolescent monkey.

## Introduction

Myopia is a common eye disorder, which is often considered to arise from complex interactions between genetic makeup and environment [1, 2]. The underlying gene—environment interactions that produce myopia are not well understood. However, over the last few decades, the prevalence and severity of myopia have risen dramatically around the world [3, 4], and in some countries of East Asia more than 80% school children now develop myopia and do so at an earlier age [5, 6]. These rapid changes in myopia incidence and prevalence imply that environmental influences may play key roles in the regulation of refractive development and in causation of the current worldwide myopia epidemic.

It is well known that the development of refraction is an active and visually guided process, called emmetropization, for matching eye length to optical power, although a genetically determined “set-point” of emmetropization may also be involved [7, 8]. Emmetropization ensures that the eye grows into and maintains the ideal refractive status (approximately emmetropic) and keeps the two eyes well matched (isometropic). Either a failure of emmetropization in infancy, or any interference with homeostasis to maintain emmetropia later in life, could result in refractive errors—including myopia, which is primarily characterized by elongation of the vitreous chamber. Evidence in various animal experiments-in chicks [9], tree shrews [10], marmosets [11] and macaque monkeys [12, 13]—indicates that optical defocus can predictably alter the normal course of emmetropization and appears to influence the refractive development. For example, when a minus lens (producing relative hyperopic defocus) is placed constantly over the eye of an infant animal, the eye elongates to compensate for the imposed hyperopic defocus until it is emmetropic with the lens in place, while the opposite is observed in the plus-lens condition. In addition, this vision-dependent mechanism has been found to be still active well beyond infancy[14]- into childhood and adolescence when the onset of “school” myopia typically occurs. Furthermore, numerous studies on the relationship between off-axis refraction and myopia have suggested that hyperopic defocus plays a critical role in the onset and progression of human myopia [15–17]. Hence, results from myopia studies based on defocus-induced myopia in animal models might be particularly relevant to the etiology of myopia in children.

Besides optic defocus, many other environmental factors, some of which can last for extended periods of time, can also affect the emmetropization process. Ambient light is one such factor. Studies have shown that a greater time spent outdoors, independent of physical activities, had a protective effect against childhood myopia.[18, 19]. Natural outdoor light (sunlight) differs from artificial indoor light in intensity and spectral composition; therefore, some researchers have speculated that relatively high intensity of outdoor light might be responsible for the protective effect of outdoor activity. The results of most animal studies, showing that high-intensity artificial lighting can slow down the progress of hyperopic defocus-induced myopia in chicks[20] and tree shrews[21], are consistent with this suggestion. These results were not confirmed in primates[22], however. In monkeys wearing monocular -3.0D lenses, Smith et al reported that exposure to artificial indoor lighting of 25,000 lux, for 150days: 6 hours each day, did not alter: the final amount of myopia, the rate at which the treated eyes compensated for the imposed defocus, or the course of recovery from the induced myopia, compared with those in lens-treated control monkeys reared in normal low-illuminance laboratory lighting. On the other hand, spectral composition has also been postulated to play a role in maintaining emmetropization, as suggested by the clinical features of several cone dysfunctions such as blue cone monochromatism, which are significantly associated with myopia[23, 24], and laboratory experiments that animals kept in red light, such as fish, chicks, and guinea pigs, developed myopic refraction compared to those kept in blue light [25–27]. Whichever factor contributes to the effect of sunlight on refractive development, these findings suggest that exposure to sunlight might prevent myopia in children. However, some epidemiological reports indicated that exposure to natural light during the early perinatal period might be associated with more moderate and high myopia [28, 29]. Taken together, these contradictory findings imply that the mechanisms by which natural light affects refractive development might be both complex and phase-dependent. The objectives of the present study were therefore to determine 1) whether natural light exposure has a protective effect on hyperopic defocus-induced myopia in infant rhesus monkeys, and 2) whether early postnatal natural light exposure causes increased risk of developing refractive error in adolescence. A preliminary report of results (1) was presented at the International Myopia Conference, IMC 2013 [30].

## Materials and Methods

### Ethics Statement

The use of the animals was approved by the Committee on the Ethics of Animal Experiments of the Sun Yat-sen University on June 30, 2010 (S1 File, Permit Number: 2010–019) and was in compliance with the ARVO Statement for the Use of Animals in Ophthalmic and Vision Research. All examinations were performed under a combination of ketamine hydrochloride/ acepromazine maleate anesthesia, and all efforts were made to minimize suffering. No monkey was sacrificed during the study.

### Animals

Sixteen healthy rhesus monkeys *(Macaca mulatta*) (LanDao Bio, GuangDong, China. Guangdong Landau Biotechnology Co. Ltd, which is eligible to breed and sale rhesus monkeys for research purposes. S2 and S3 Files) were used in the study. All the monkeys were reared at the Ophthalmic Animal Laboratory for Zhongshan Ophthalmic Center of Sun Yet-sen University under a 12:12 light—dark cycle, with lights on at 8 AM and off at 8 PM. Each animal was housed individually in a fine steel cage, which allowed practically all of the incident light to reach the animal. The laboratory room is around 16 square meter and is equipped with temperature and humidity control system which keep the room temperatures 25°C±2°C and the relative humidity at 45%±5%. The animals were housed in cages individually with free access to food and water. The steel cages used indoors were 800mm×770mm×1140mm, while those outdoors were 400mm×400mm×500mm and were easy to move. Eight of them were obtained at age of 20–30 days and were reared until they grew into adolescence, approximately at 3 years of age. Another eight 3 year-old monkeys reared in the same laboratory were employed to provide control data on normal development in puberty.

The feeding regimens have been reported by elsewhere[13]. Specifically, the infant monkeys had been bottle-feeding infant formula five to six times per day until they were 4 months old. When the monkeys grew up and could eat solid foods independently, the diet which ensured good nutrition for all monkeys included primate feed, vegetable meal and fruit meal.

To alleviate suffering of the animals during the experimental period, monkeys were anesthesia for every examination. Anesthesia was accomplished by intramuscular injection a combination of ketamine hydrochloride + acepromazine maleate (10 mg/kg + 0.2 mg/kg) and topical application of one drop of 0.4% oxybuprocaine hydrochlorid. In addition, one to two drops of 0.3% tobramycin were used after each examination to avoid ocular infection.

### Experimental Design

#### Experiment A

All of the infant monkeys were fitted with lightweight helmets, provided by Dr. Earl L. Smith 3rd (College of Optometry, University of Houston, Houston, TX), at approximately 20–30 days of age, incorporating a -3.0D spectacle lens over the right eye and a zero-power lens over the left eye for hyperopic anisometropia. The lenses were worn continuously, except when removed for cleaning or eye examination, for about 190 days. The details of the maintenance of the lens have been described elsewhere[13].

After wearing the lens, the animals were randomly divided into two groups (n = 4 monkeys in each group): A) AL (artificial lighting) monkeys were kept under fluorescent lamps (YZ28RR16, 28W, color temperature = 6500K). Illuminance at the level of the monkeys’ eyes was 100-200lux, and the main output wavelengths were 453nm, 545nm and 611nm. B) NL (natural lighting) monkeys were exposed to natural outdoor light for 3 hours, from 11:00 to 14:00 each day, and were housed in the same indoor setting as the AL monkeys during the rest of the light phase. As the weather changed, the intensity of the sunlight was not stable. A photometer (1332A, TES, Taiwan, China) was used to determine the illuminance at the level of the animals’ eyes at 30-minutes intervals, every day of treatment. When it was cloudy, the light intensity varied from 6,000 to 10,000 lux; but when it was sunny, the illuminance could reach 60,000 to 70,000 lux. Throughout the course of the experiment, the average highest light intensity ranged from 25,000 to 40,000lux. In addition, the spectrum of the sunlight is across most of the electromagnetic spectrum, spanning a range of 200nm to about 1mm.

#### Experiment B

To examine whether the early life exposure to sunlight would associate with the onset of refractive errors, all of the helmets were removed after the last biometry measurements of experiment A, at approximately 215±3 days of age. Then, the monkeys regained unrestricted vision and were raised in the same laboratory room (without exposure to outdoor light) until they were about 3 years old (1185 ± 3 days of age). That age in monkeys can be considered to be equivalent to 12 years of age in humans, well into the age range when school myopia frequently develops in children. Comparison data were available from eight 3-years-old monkeys reared from birth with unrestricted vision, under the standard indoor lights and conditions described above.

### Ocular Biometry

The spherical equivalent refractive error, corneal curvatures, and axial dimensions of each eye of each monkey were measured before lens-wearing (at 23±3 days of age), monthly during the lens-wearing treatment period, and at the age of puberty (at 1185+3 days of age). To make these measurements, the monkeys were anesthetized with a combination of ketamine hydrochloride + acepromazine maleate (10 mg/kg + 0.2 mg/kg, respectively, intramuscular). Retinoscopy was performed by two experienced optometrists using a streak retinoscope and hand-held trial lens, after topical application of three drops of 0.5% tropicamide for cycloplegia. Mean spherical equivalent spectacle-plane refractive correction was recorded for a given eye’s refractive status. Corneal curvature was assessed using a handheld videotopographer (Vista; EyeSys, Houston, TX). The axial dimensions were measured by A-scan ultrasonography (AXIS-II; Quantel Medical Inc., Clermont-Ferrand, France); the 11-MHz transducer was placed in direct contact with the cornea, following topical anesthesia with one drop of 0.4% oxybuprocaine hydrochloride. The details of these determinations have been reported previously[12]. All the examiners in our experiment were blinded to the animal groups and each other’s findings.

### Statistical Analysis

Mann-Whitney U test were used to compare the median of refraction and vitreous chamber depth between the two groups at the start of lens-rearing treatment. Since the lens-rearing regimen altered emmetropization process in both eyes of every animal in experiment A, each individual eye was recorded and treated as an independent sample. However, considering the emmetropization in the two eyes of the animals may not be totally independent, a linear mixed model, which is a useful approach allowing correlation in observations, was used to compare the longitudinal change of refractive errors and vitreous chamber depth in the two groups. To further assess the effect of different ambient light on defocus-induced myopia, all data are presented as mean intrerocular differences, i.e. differences between treated eye and fellow eye. Independent-samples *t*-tests were used to compare intraocular differences between NL group and AL group at the start and the end of lens-rearing period respectively. In experiment B, a paired-samples *t* Test was used to assess the interocular differences. One-way ANOVAs were employed to analyze interocular inference of measured ocular parameters among the three groups, and least-significant difference tests were further applied in post hoc multiple Comparisons. Pearson’s correlation analysis was used to examine the relationship between refractive status and vitreous chamber depth. All measurements were analyzed on computer (SPSS 11.0, IBM, NY, USA).

## Results

### The effect of sunlight exposure on lens-induced myopia in infant rhesus monkeys

**Fig 1:**
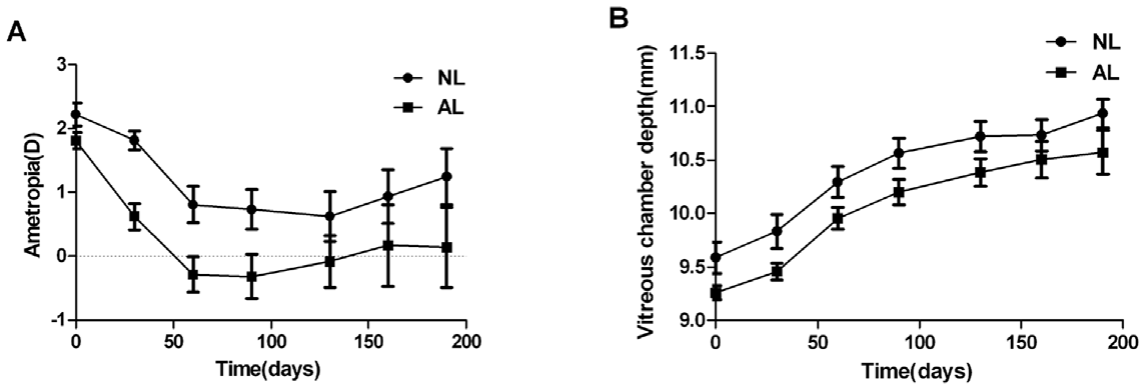
Changes in refraction and vitreous chamber depth of infant monkeys in AL and NL. (A) Spherical-equivalent refractive error and (B) vitreous chamber depth plotted as a function of time of lens wearing for infant monkeys reared under artificial lighting (AL) and natural lighting (NL) respectively. Each point represents the mean±SE (bar) of 8 eyes from the same group.

The data in the both eyes of each animal were collected to observe a general effect of sunlight on refractive development. Fig 1 shows the longitudinal changes in the mean spherical equivalent refractive errors and vitreous chamber depth in the two groups. Each point presents the mean measurement of 8 eyes from the same group. As shown in Fig 1A, exposure to sunlight for 3 hours per day over 190 days reduced the development of myopia in comparison to that seen in the infant monkeys reared under artificial light. Refraction in the NL group was more hyperopic than that in the AL group during the lens-rearing period. A linear mixed model for repeated measure analysis found significant difference in changes of refractive error over time between the two groups (F = 5.726, *P* = 0.032), whereas no statistical difference was found in the rate of vitreous chamber elongation (F = 3.768, *P* = 0.074). However, because the average value of vitreous chamber depth was greater in the NL group at the start of treatment (Z = -1.997, *P* = 0.046), the same amount of refraction need more change in axial length (Fig 1B). In this respect, the difference in refraction between the two groups is correlated with the difference in vitreous chamber depth. There was no statistically significant difference in refractive errors (0D: Z = -1.276, *P* = 0.202; 190D: Z = -1.314, *P* = 0.189) and corneal power (0D: Z = -1.504, *P* = 0.132; 190D: Z = -0.694, *P* = 0.487) between both groups at the start and the end of lens-rearing treatment (Table 1).

**Table 1:** Refractive Errors and Ocular Parameters at the start and the end of lens-rearing experiment.

		Ametropia(D)		ACD(mm)		LT(mm)		VCD(mm)	
	Eye	Day0	Day190	Day0	Day190	Day0	Day190	Day0	Day190
NL group	Right	**+2.19±0.55**	**+0.84±1.57**	**2.64±0.20**	**2.82±0.19**	**3.88±0.13**	**4.24±0.12**	**9.58±0.43**	**11.03±0.44**
N = 4	Left	**+2.20±0.54**	**+1.66±0.84**	**2.63±0.18**	**2.93±0.20**	**3.83±0.13**	**4.15±0.06**	**9.60±0.47**	**10.95±0.35**
AL group	Right	**+1.81±0.38**	**-0.50±2.33**	**2.39±0.13**	**2.77±0.10**	**3.96±0.05**	**4.26±0.18**	**9.25±0.24**	**10.79±0.69**
N = 4	Left	**+1.81±0.38**	**+0.78±0.93**	**2.36±0.16**	**2.89±0.12**	**3.96±0.09**	**4.23±0.18**	**9.27±0.14**	**10.35±0.41**

**Legend: D, Dioptor; K, Corneal power; ACD, Anterior chamber depth; LT, Lens thickness; VCD, Vitreous chamber depth; Values given as the mean±SD; Right, Minus lens wearing eye; Left, Contralateral eye**

**Fig 2:**
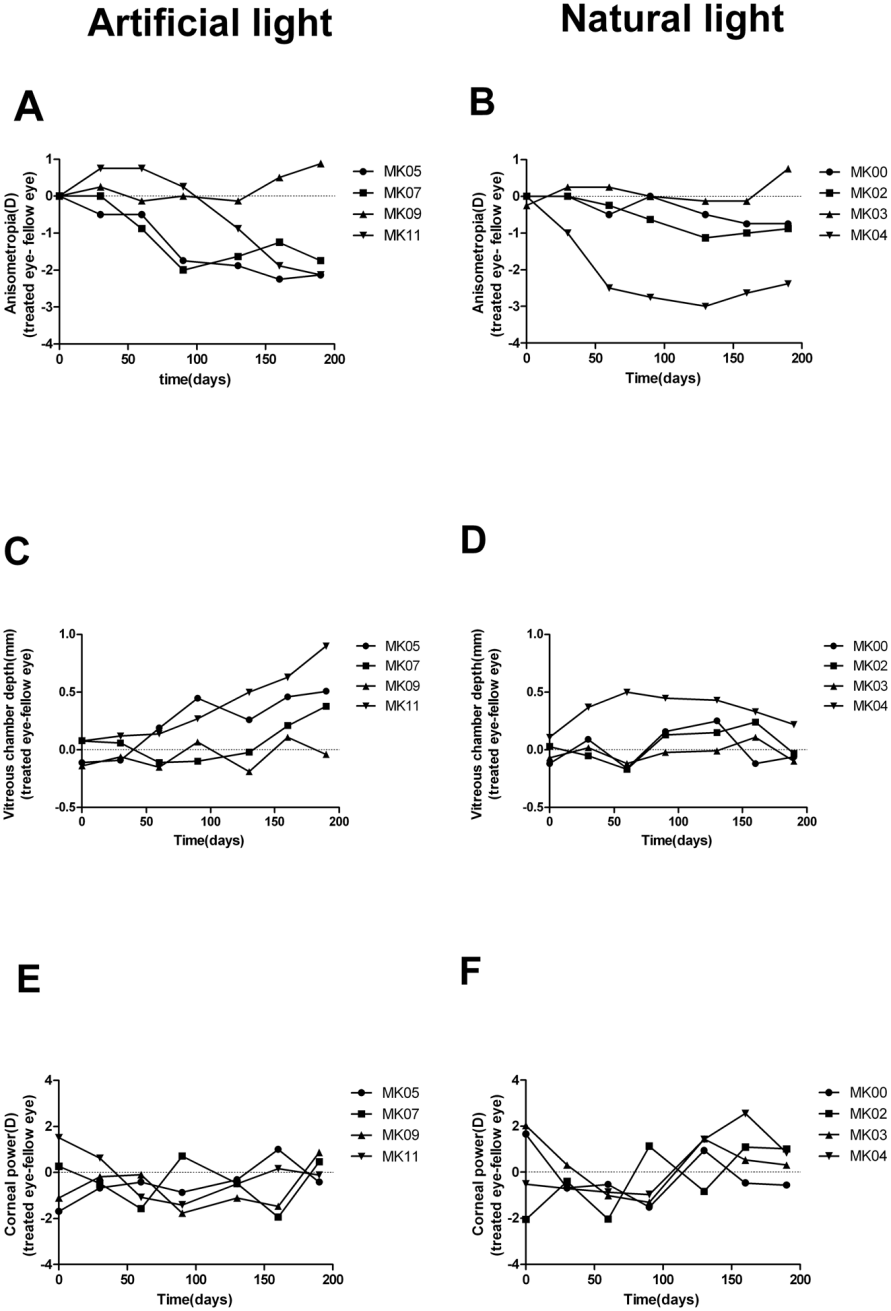
Interocular differences in refraction and ocular parameters of infant monkeys in AL and NL. The interocular differences in spherical-equivalent refractive error (A-B), vitreous chamber depth (C-D) and corneal power (E-F) plotted as a function of time of lens wearing for individual infant monkeys reared under natural lighting (left panel) and artificial lighting (right panel) respectively.

At the start of lens-rearing treatment, the refractive status, corneal power, and vitreous chamber depth of the two eyes in each infant monkey were very similar. There were no between-group differences in these ocular parameters (NL group versus AL group: anisometropia: 0.07±0.13D vs 0.00±0.00D, *t* = -1.0; *P* = 0.356, vitreous chamber depth: -0.16±0.10mm vs 0.02±0.12mm, *t* = 0.127, *P* = 0.903; corneal power: 0.28±1.91D vs -0.25±1.44D, *t* = 0.445, *P* = 0.672). Over the next 190 days of unilateral hyperopic-defocus regimen, three of four infant monkeys under artificial lighting developed myopic anisometropia more than 2.0D, although the refractive errors in individual monkeys fluctuated over time and reached maximum anisometropia at variable times—ranging from 90 to 150 lens-rearing days (Fig 2A). However, as illustrated in Fig 2A, the refractive errors of both eyes in animal MK09 in the AL group remained isometropic during most of the treatment period, reaching only a minor hyperopic anisometropia at the end. In contrast, Fig 2B shows the development of anisometropia in monocularly lens-reared monkeys exposed to sunlight. In this group, only animal MK04 exhibited more than -2.0D anisometropia, approximately 60 days after the commencement of lens rearing, while the other three monkeys maintained less than 1D myopic anisometropia throughout the lens-wearing period. However, because there were outliers in both groups, and because the sample sizes were small, the differences between the groups did not reach statistical significance by the end of treatment (NL group versus AL group: anisometropia: -0.82 ± 1.27D vs -1.28 ±1.45D, *t* = 0.483; *P* = 0.646).

Previous studies have shown that negative lens-induced myopia in animal models is generally axial in nature. Fig 2C and 2D compare the interocular differences in vitreous chamber depth of each monkey over time, under sunlight and artificial lighting, respectively. As expected from the imposed anisometropia, three monkeys in the AL group (MK05, 07 & 11) developed more than 2.0D anisometropia and showed increased differences in vitreous chamber depth, whereas the interocular differences in animal MK09 changed very little during the entire lens-wearing period. In contrast, in most of the sunlight-exposed infant monkeys (MK00, 02, & 03), the differences in vitreous chamber depth were quite stable throughout the experimental procedure, but the animal MK04 exhibited a significantly increased interocular difference in vitreous chamber depth. Although the overall trends for change in vitreous chamber depth were clearly different for 3 of the 4 animals in the two treatment groups, the differences at the end of the monitoring period were not statistically significant (NL group vs. AL group:-0.07±0.14mm vs.0.44±0.39mm; *t* = -2.079; *P* = 0.083; 190 days), possibly for the same reason as that for the development of anisometropia (previous paragraph).

The interocular differences in corneal power in both groups (Fig 2E and 2F) were not significantly different at the end of the experiment (NL group vs. AL group: 0.40±0.71D vs.0.51±1.03D; *t* = 1.452; *P =* 0.197;190 days).

### The effect of sunlight exposure during infancy on refractive development in adolescence

In experiment B, eight 3-year-old monkeys with unrestricted vision since birth were used as control group, which were reared with the same 12/12 light-dark cycle under ordinary artificial lighting (200lux). Assessment by a paired-sample *t* test indicated that refractive states (OD vs OS:-0.03±1.97D vs -0.08±1.90D, *t* = 0.469, *P* = 0.654), vitreous chamber depth (OD vs OS: 12.15±0.66mm vs 12.14±0.65mm, *t* = 0.279, *P* = 0.788) and corneal power (OD vs OS: 50.72±1.66D vs 50.78±1.72D, *t* = -0.196, *P* = 0.850) for the right and left eye of all control animals were well matched. Therefore, we analyzed only the data of the right eyes in this control group. Although the mean refractive errors of monkeys in the artificial lighting group seemed to be more myopic in comparison to monkeys in the other two groups (Table 1), One-way ANOVA did not confirm any significant differences in refraction (*F* = 1.183, *P* = 0.35), vitreous chamber depth (*F* = 2.173, *P* = 0.111), or corneal power (*F* = 1.714, *P* = 0.188) among the three groups (Table 2).

**Table 2:** Refractive Errors and Ocular Parameters at the age of three.

	Eye	Ametropia(D)	K(D)	ACD(mm)	LT(mm)	VCD(mm)
Control, n = 8	**Right**	**-0.03±1.97**	**50.72±1.66**	**3.71±0.33**	**3.89±0.16**	**12.15±0.66**
	**Left**	**-0.08±1.90**	**50.78±1.72**	**3.58±0.29**	**3.95±0.15**	**12.14±0.65**
NL group, n = 4	**Right**	**-0.19±1.01**	**48.81±1.21**	**3.39±0.22**	**3.88±0.17**	**12.76±0.61**
	**Left**	**0.03±0.65**	**49.17±1.51**	**3.56±0.22**	**3.95±0.07**	**12.73±0.43**
AL group, n = 4	**Right**	**-1.81±1.92**	**50.65±1.44**	**3.36±0.18**	**3.88±0.33**	**12.23±0.42**
	**Left**	**-1.22±1.48**	**50.37±1.13**	**3.34±0.24**	**3.91±0.43**	**11.83±0.47**

**Legend: D, Dioptor; K, Corneal power; ACD, Anterior chamber depth; LT, Lens thickness; VCD, Vitreous chamber depth; Values given as the mean±SD.**

**Fig 3:**
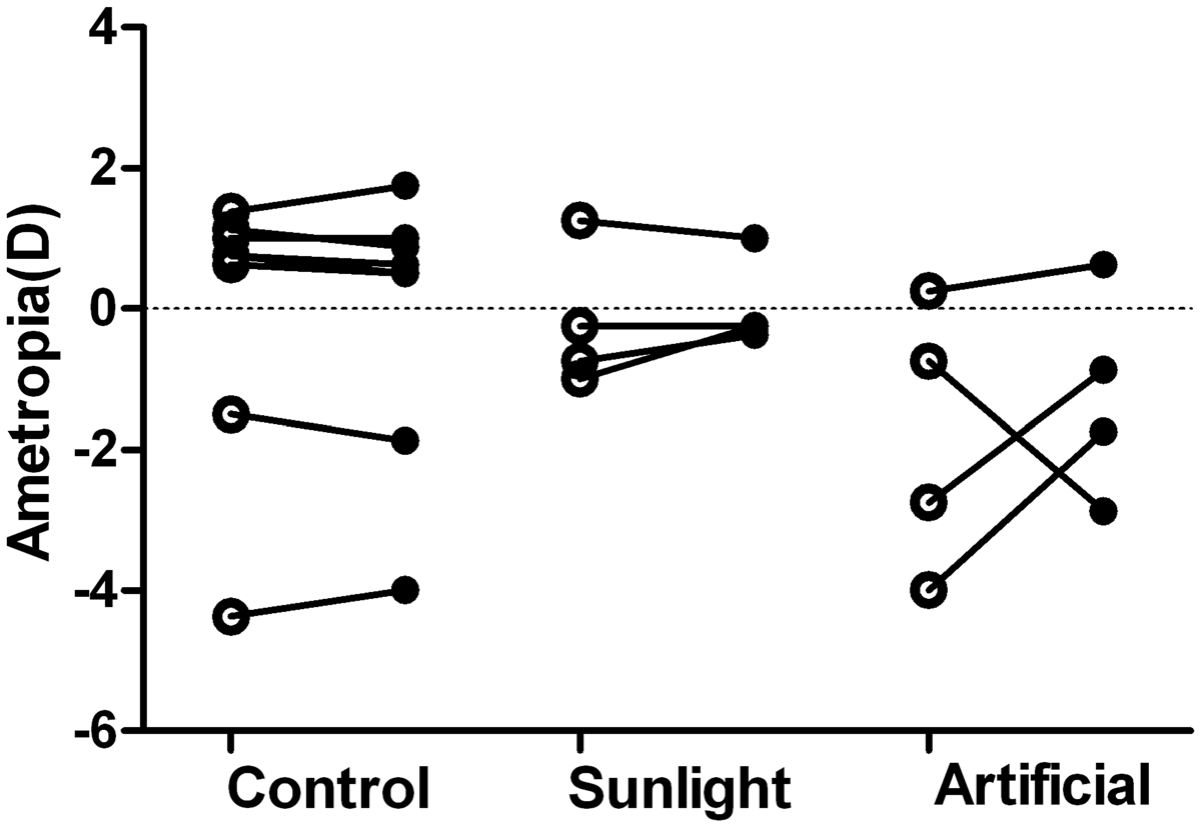
Refractive errors of adolescent monkeys in the three groups. Spherical-equivalent refractive error measured at adolescence for the right (○) and left (●) eyes of individual monkeys from the three groups: (1) aged-matched normal group (Control), (2) NL group (Sunlight),(3) AL group (Artificial). The lines connect the right and left eyes of individual monkeys. The right eyes in the AL and NL group were treated with minus lenses early in life and the left eyes were contralateral eyes.

However, it is interesting that the absolute anisometropia (i.e. the difference of maximum and minimum refraction) in 3-year-old monkeys that were treated by monocular hyperopic-defocus during infancy under artificial lighting was significantly greater and more myopic than that in monkeys in the NL group (AL vs NL: -1.66±0.87D vs -0.22±0.44D; P = 0.002) or the 3-year-old control group (AL vs Control: -1.66±0.87D vs -0.05±0.85D; P<0.0001) at the same 3-year-old end-point. As illustrated in Fig 3, which shows the ametropia for the right eye and left eye of each animal at the age of 3 years, three of four animals in the AL group demonstrated absolute myopic anisometropia more than 1.75D: two of these developed relative myopia in the originally lens-induced eye, but the other developed myopia in the fellow eye. By contrast, the results for lens-induced monkeys reared under sunlight and those for control monkeys were not different (NL vs Control: -0.22±0.44D vs -0.05±0.85D; P = 0.884): the absence of anisometropia over 0.75D indicated that the refractive development in the two eyes of these animals was well coordinated.

**Fig 4:**
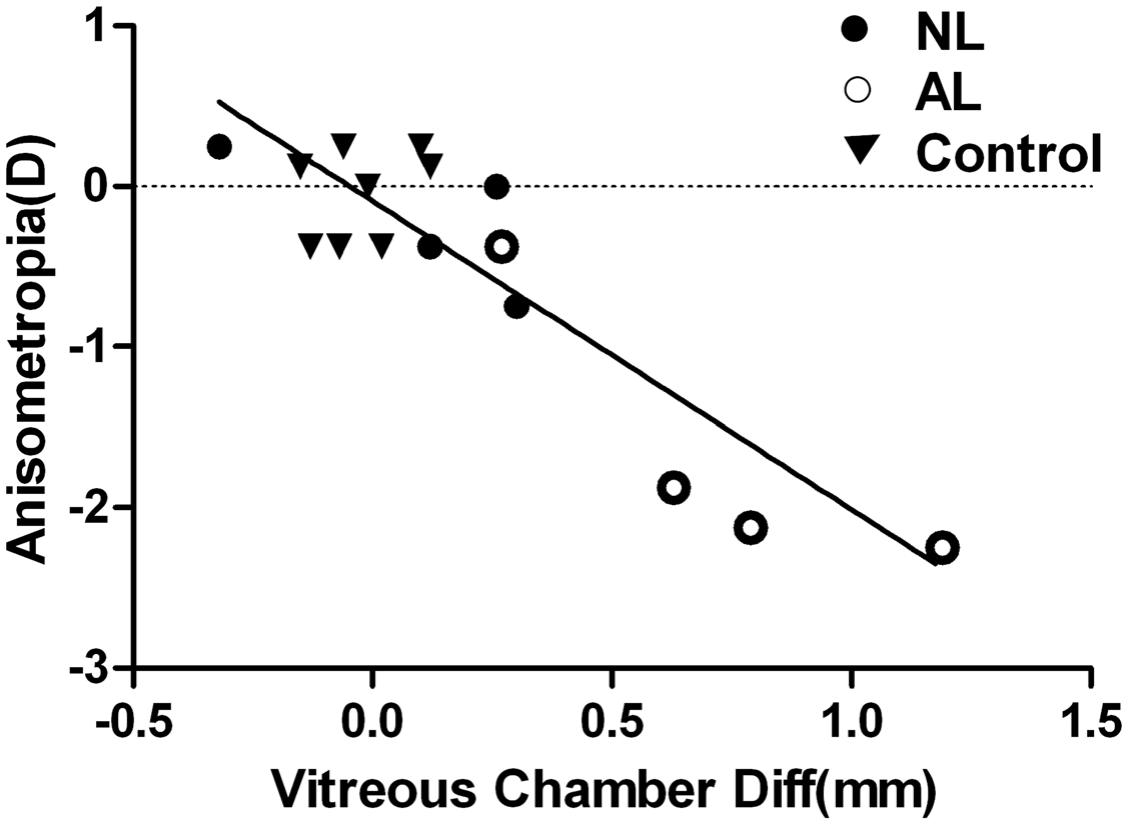
Relationship between anisometropia and interocular differences in vitreous chamber depth of adolescent monkeys in the three groups. Absolute interocular differences in spherical-equivalent refractive error plotted as a function of interocular differences in vitreous chamber depth for individual animals. Solid line: best-fitting regression line (y = -1.921x-0.091; r^2^ = 0.769).

**Fig 5:**
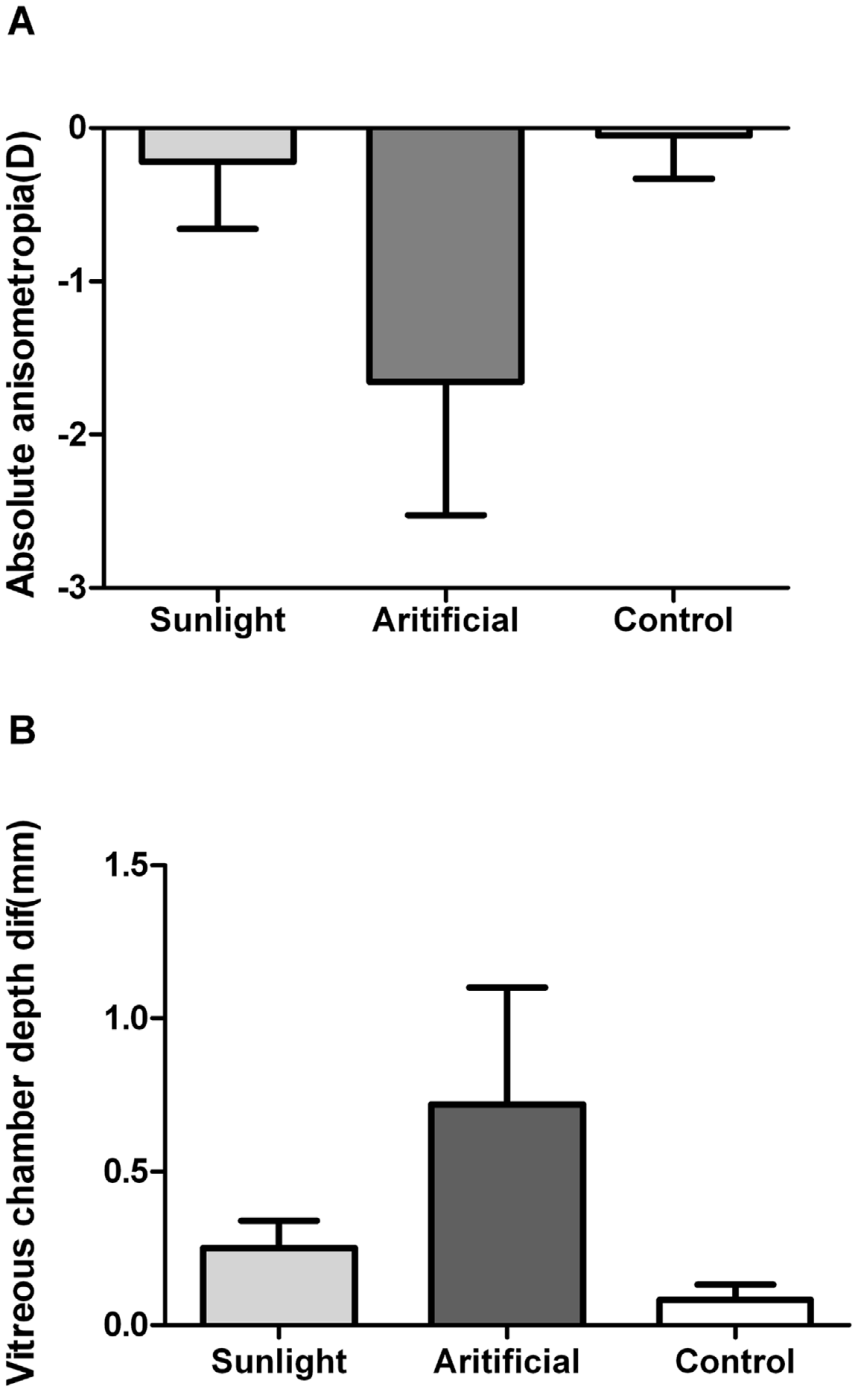
Anisometropia and interocular differences in vitreous chamber depth of adolescent monkeys in the three groups. Interocular differences in Spherical-equivalent refractive error (A) and vitreous chamber depth (B) measured for adolescent monkeys from three groups: (1) aged-matched normal group (Control), (2) NL group (Sunlight), (3) AL group (Artificial). Data columns represent the means±SE (bars).

In all groups of monkeys, absolute myopic anisometropia was associated with a difference (excessive increase) in vitreous chamber depth (Fig 4): r^2^ = 0.769, P<0.0001). Furthermore, Fig 5A and 5B represent the mean values as well as the reciprocal relationship of the interocular difference in refractive errors and vitreous chamber depth from all three groups. One-way ANOVA indicated a statistically significant difference across groups with respect to interocular differences in vitreous chamber depth (F = 14.784, P<0.001), with this parameter being larger in the AL group than in the NL (P = 0.004) and control groups (P<0.001). However, the interocular differences in vitreous chamber depth did not differ significantly between adolescent monkeys that had been treated by lenses and sunlight early in life, and those reared under indoor light with unrestricted vision since birth (P = 0.178).

## Discussion

A number of recent epidemiological studies have suggested that greater time spent outdoors has protective effect on the incidence and progression of school myopia [18, 31–34]. Most importantly, this positive effect is not associated with physical activity and is also independent of near work, parent myopia, and ethnicity, which have been proposed as myopiagenic factors. What mechanism can explain this protection of outdoor experience against myopia is unclear, but some environmental factors have been speculated, including: the relatively flat dioptric topographies of outdoor scenes, the high level of ambient lighting encountered outdoor, and the different spectral component of sunlight vs typical indoor light. We assume that exposure to sunlight *per se*, rather than other factors, is responsible for the anti-myopia effect of time spent outdoors, possibly due to a synergy between high lighting levels and an abundance of short-wavelength light. Consequently, in the first part of this study we compared refractive development and defocus-induced myopia in monocular lens-wearing infant rhesus monkeys, reared under ordinary artificial indoor lighting, with that in monkeys exposed to sunlight, to investigate the effects of sunlight on refractive development.

In order to observe the general effect of sunlight on refractive development, we pooled the data from both eyes of the animals in the same group. Recent studies demonstrated that unilateral lens treatment can influence eye growth and refractive development also in the untreated contralateral eye [35, 36]. But this yoking effect was very small under the -3D lens-rearing paradigm[13, 22]. Thus, it is valid that we used a linear mixed model to analyze the data, which is appropriate for repeated measure designs allowing correlation in data. The results found that the rearing of monkeys with monocular hyperopic-defocus spectacles under sunlight for only 3 hours per day for 190 days (the duration of effective outdoor exposure suggested by the study of Rose et al[18]) induced significantly more hyperopia than those under artificial lighting, which is now considered to correlate with a lower incidence of myopia. Moreover, we observed different patterns over time, involving compensation to imposed anisometropia between the two groups, as 75% AL animals developed myopia more than -2.0D, whereas, most NL animals remained isometropic. Nevertheless, this difference was not statistically significant. Finally, these changes in refractive error were correlated with the rate and final extent of vitreous chamber growth, as reported previously in other animal studies. Overall, these findings provide support for the conclusion that daily exposure to natural light (sunlight) tends to inhibit hyperopic-defocus induced myopia in infant rhesus monkeys.

There are numerous ways in which sunlight affect the development of refraction or prevention of myopia. As mentioned above, optical defocus has been identified as one of regulators in emmetropization, which is actively guided by visual processes—mainly in the retina. Nonetheless, other visual stimulus properties—including light intensity, spectral components, spatial and temporal contrast and perhaps others that are still not recognized,—can modulate the process. Since sunlight is different from artificial light used in our study being up to 100 times higher luminance and richer in shorter wavelengths, it is likely that both of these factors contribute to the beneficial effects of sunlight on refractive development. With respect to high ambient light intensity, rearing chicks with 10,000lux lighting delayed the time of emmetropization and conserved hyperopia [37], and a regimen of 15,000lux artificial lighting slowed the rate of compensation to lens-induced myopia in chicks [20] and tree shrews[21]. Similarly, in monocularly form-deprived rhesus monkeys, the non-deprived fellow eyes of monkeys reared under high-intensity artificial lighting, averaging 25,000lux for 6 hours per day, were more hyperopic than normal[38]. These findings, along with our results, support an anti-myopia effect of high luminance *per se*. However, in another study, Smith et al. observed that the same illumination protocol had no effect on the compensation for and recovery from myopia induced by -3.0D [22].

Are there apparent differences between the study of Smith et al. (2013) and the present one that might explain the difference in results? Yes, clearly there were several significant differences between conditions in these two studies. One difference was the mean light level: the illuminances in Smith’s study averaged 25,000lux whereas the illuminance of sunlight in our study varied widely, from 6,000lux to 70,000lux, and the mean illuminance was higher, from 25,000 to 40,000lux. Some evidence has indicated that higher light intensities have specific effects on ocular growth: for example, Asbby et al.[32] have reported a stronger retardation of form-deprived myopia in chicks exposured to sunlight than in those exposed to 15,000lux artificial lighting, possibly because of the greater intensity of outdoor light. Another major difference between sunlight and artificial lighting is spectral composition: although the metal halide lamps used in an earlier study emit strongly throughout the visual spectrum, this source is stronger in middle and long length wavelengths but weaker in short wavelengths, while typical outdoor sunlight contains a preponderance of blue light [39]. Clinical research studies have shown that patients with either protanopia or enhanced S-syndrome may be more hyperopic than the average trichromat, suggesting that tipping the balance towards activation of S-cones could be protective against myopia. [40, 41]. Similar conclusions were drawn by Kroger and his colleagues, who reported that the use of green paper selectively absorbing long wavelengths may reduce the myopiagenic effects of near work[42]. Finally, stronger evidence comes from studies of chicks which developed hyperopia when reared in light consisting mainly of shorter wavelengths, but developed myopia when raised in light mainly of longer wavelengths[43, 44]. Therefore, the difference between results of the present study and those reported earlier, in particular the protective effect of sunlight against lens-induced myopia in rhesus monkeys could be due to both the higher-light intensity and the preponderance of short wavelengths in sunlight in the present study, but it is impossible to differentiate between these two factors in our experiment.

However, not all evidence supports the hypothesis that exposure to sunlight could prevent the development of myopia. For instance, a large study in Israel including 276,911 adolescents found that young adults born in the summer had a higher prevalence of moderate and severe myopia in comparison to those born in the winter, suggesting that increased exposure to sunlight early in life is a risk factor for developing myopia later in life[28]. Two subsequent studies in the U.K and U.S.A in adults of a wide age range and in children aged 1–3 months, respectively, produced similar results; the authors proposed that seasonal differences in outdoor light exposure (longer time spent outdoors and more intense sunlight during the warmer months) were causally related to the significantly different incidence of myopia in different birth seasons [29, 45]. To test this hypothesis, in the second part of this study we observed refraction and ocular parameters of the eight lens-treated monkeys when they were 3 years old. Unexpectedly, we found that imposing hyperopic anisometropia in rhesus monkeys during infancy, under artificial indoor lighting, caused myopic anisometropia when these animals grew into puberty, and that this was associated with an increase in the difference in vitreous chamber depth between the two eyes. In contrast, adolescent monkeys that had been given outdoor experience during infancy did not develop myopic anisometropia with excessive axial elongation. Thus, in our monkeys, the risk of developing myopic anisometropia later in life could be reduced by daily exposure to natural light early in life.

It has been documented in a variety of animals that the induced anisometropia produced by unilateral negative lens-treatment early in life can be eliminated after the restoration of unrestricted vision [38, 46, 47]. However, there is little evidence whether those animals could keep the balance of refractive status in the two eyes for a long time, which is another important aspect of normal emmetropization (i.e. achieving isometropia). Although longitudinal data after restoration of unrestricted vision were not available, other than monocular hyperopic-defocus there was nothing in the history of the AL monkeys to distinguish them from the eight animals in the untreated indoor control group. In this respect, our findings suggested that alteration of visual input to one eye during infancy, e.g., by hyperopic defocus, might have potential to permanently impair the ability to maintain isometropia. Supporting our speculation, an early study—in which more than half of 12 unilaterally diffuser-reared monkeys failed to stay isometropic after recovery from form-deprived myopia—indicated that unilateral alteration of visual input during infancy could produce chronic interocular asymmetry in the eye’s response to optical defocus.[48]. This might be explained in terms of visual development: The development of the visual system begins in the early postnatal days and matures during early childhood. There are different sensitive periods at various levels of visual function, during which detrimental visual experience can disrupt the normal developmental process and induce long-lasting response deficits in visual function. Several lines of evidence show that the defocus differentiation and visual signal transduction processes involved in regulating refraction development and ocular growth are located mainly, if not exclusively, in the retina[49–52], where the sensitive periods of visual development are relatively short. For example, in rhesus monkeys, development of the basic spectral sensitivity functions of rods and cones ends at 3 months and 6 months of age, respectively[53]. Therefore, in our study the unilateral lens-rearing regimen was likely to cover the sensitive period of retinal function that is critical for accurate response to optical defocus, and this resulted in interocular imbalance of refractive state in later life.

On the other hand, the same unilateral lens-rearing protocol under sunlight did not cause significant myopic anisometropia as those monkeys grew into adolescence. Why was that? Again we suggest that this could be due to the difference in luminance and spectral component between the sunlight and the artificial light used in the study: The fluorescent light lacks variety in luminance intensity and has discontinuous spectral makeup, which is a mixture of three wavelengths including 453nm, 545nm and 611nm, while the light level of sunlight changed with time and weather, and the spectrum contains all wavelengths of visible light. Changes in both luminance and color contrast have been demonstrated to contribute to the discrimination process that the eye determines the sign of defocus [54–56], though this exact mechanism is unknown yet. For instance, the compensatory response to lens-rearing in chicks was more accurate in white light than in monochromatic light, indicating that multiple wavelengths might be involved in detecting the defocus [26]. Furthermore, the results of several animal experiments have implied that temporal changes in luminance on the retina have influenced the set-point of emmetropization [57–59]. Combined with these findings, the fact that the adolescent monkeys in the AL group developed interocular asymmetry of refraction, whereas those in NL group did not, supports the hypothesis that luminance and color information from visual stimulation during infancy might be necessary for the development of emmetropization, and that more adequate information might be more accurate response to optical defocus. If that is the case, and if the sensitive period for response to defocus was indeed included in our treatment course (as we reasonably conclude), the retinas of NL monkeys were more likely to develop normal visual function and keep balance in the two eyes in their later life, because of full spectrum and much more changes in luminance in sunlight during the early treatment period.

In conclusion, our study demonstrates that exposure to natural light has an effect to reduce hyperopic defocus-induced myopia, supporting a role for sunlight in protection against myopia by outdoor activity in school-aged children. Further, we present evidence indicating that exposure to sunlight early in life promotes normal emmetropization in later in life, and thus lower the risk of myopic anisometropia in adolescent monkey. However, there are limitations in our study (e.g. small size of animals, lack of the more details in lighting parameters), and there is still much to be learned about the mechanism(s) by which exposure to sunlight prevents the development of myopia, further studies with larger scale and more details in basic lighting parameters are needed in the future.

## Supporting Information

S1 File Ethics Committee Review. (PDF)

S2 File Breeding License. (PDF)

S3 File Organization Code Certificate. (PDF)

S4 File Production License. (PDF)

S5 File Animal Use Certificate. (PDF)
