# Supplementary material for: Exposure to Sunlight Reduces the Risk of Myopia in Rhesus Monkeys
Source: PLoS One. 2015 Jun 1;10(6):e0127863. doi: 10.1371/journal.pone.0127863 (PMC4451516; doi:10.1371/journal.pone.0127863)
Supplement: S1 File — (PDF) [file pone.0127863.s001.pdf]

中山大学中山眼科中心
动物实验伦理委员会审查报告

Ethics Committee review of animal experiments Zhongshan Ophthalmic Center，Sun Yat-sen University

实验名称 (Study title)	幼年恒河猴近视眼动物模型的建立及机制研究		
项目来源 (Project sources)	国家自然基金		
项目申请人 (Applicant)	丁辉	受理编号 (Acceptance number)	2010-019
审查形式 (Auditing)	✓ 会议/通讯 (Meeting/Communication)	审查时间 (Processing time)	2010-6-30

实验动物管理和伦理委员会签名(Signature of Institutional Animal Care and Use/ Ethics Committee Members)：

葛坚	中山大学中山眼科中心<br>Zhongshan Ophthalmic Center, Sun Yat-sen University	
王智崇	中山大学中山眼科中心<br>Zhongshan Ophthalmic Center, Sun Yat-sen University	
吴开力	中山大学中山眼科中心<br>Zhongshan Ophthalmic Center, Sun Yat-sen University	
张清炯	中山大学中山眼科中心<br>Zhongshan Ophthalmic Center, Sun Yat-sen University	张清炯
张秀兰	中山大学中山眼科中心<br>Zhongshan Ophthalmic Center, Sun Yat-sen University	
李永平	中山大学中山眼科中心<br>Zhongshan Ophthalmic Center, Sun Yat-sen University	李永平
钟兴武	中山大学中山眼科中心<br>Zhongshan Ophthalmic Center, Sun Yat-sen University	钟兴武
黄韧	广东省实验动物监测所<br>Guangdong Laboratory Animals Monitoring Institute	
黄冰	中山大学中山眼科中心<br>Zhongshan Ophthalmic Center, Sun Yat-sen University	黄冰
刘利鸽	中山大学中山眼科中心<br>Zhongshan Ophthalmic Center, Sun Yat-sen University	刘利鸽
黎韦华	中山大学中山眼科中心<br>Zhongshan Ophthalmic Center, Sun Yat-sen University	黎韦华

审查结果(Results of the review)：

该实验方案符合动物福利伦理要求，通过动物实验伦理审查，准予开展动物实验。

中山眼科中心实验动物管理和伦理委员会

Institutional Animal Care and Use/ Ethics Committee Zhongshan Ophthalmic Center

伦理委员会主任委员签章（Signature of Ethics Committee Director）：葛坚

时 间(time)：2010.6.30

中山大学中山眼科中心 实验动物管理和伦理委员会
