# Supplementary material for: Exposure to Sunlight Reduces the Risk of Myopia in Rhesus Monkeys
Source: PLoS One. 2015 Jun 1;10(6):e0127863. doi: 10.1371/journal.pone.0127863 (PMC4451516; doi:10.1371/journal.pone.0127863)
Supplement: S2 File — (PDF) [file pone.0127863.s002.pdf]

国家重点保护野生动物
驯养繁殖许可证

广东蓝岛生物技术有限公司：　　　　　　　　　　粤发 驯繁（2009－001－）号

经审核，允许驯养繁殖国家重点保护野生动物，特发此证。

（准许驯养繁殖种类见副本）　　　　　　发证机关

广东省林业局

二〇〇九年 二月 十三日

广东蓝岛生物技术有限公司
