# Supplementary material for: Exposure to Sunlight Reduces the Risk of Myopia in Rhesus Monkeys
Source: PLoS One. 2015 Jun 1;10(6):e0127863. doi: 10.1371/journal.pone.0127863 (PMC4451516; doi:10.1371/journal.pone.0127863)
Supplement: S3 File — (PDF) [file pone.0127863.s003.pdf]

中华人民共和国
组织机构代码证

（副 本）

代　　码：7 4 6 2 9 5 8 8 - 8

机构名称：广东蓝逸生物技术有限公司

机构类型：企业法人（法定代表人：韩日畴）

地　　址：广东省广州市萝岗区九龙镇红卫广华路３３号１０１房

有 效 期：自2012年10月11日至2015年09月24日

颁发单位：广州市质量技术监督局

登 记 号：组代管440100-3648544

说　明

1. 中华人民共和国组织机构代码是组织机构在中华人民共和国境内唯一的，始终不变的法定代码标识。《中华人民共和国组织机构代码证》是组织机构法定代码标识的凭证，分正本和副本。
2. 《中华人民共和国组织机构代码证》不得出租、出借、冒用、转让、伪造、变造、非法买卖。
3. 《中华人民共和国组织机构代码证》登记项目发生变化时，应向发证机关申请变更登记。
4. 各组织机构应当按有关规定，接受发证机关的年度检验。
5. 组织机构依法注销、撤消时，应向原发证机关办理注销登记，并交回全部代码证。

中华人民共和国　国家质量监督检验检疫总局签章

有效期内每年10月10日前30日内年检
2015年09月24日前30日内换证

年检记录

2013年已年检 8120 6537	年 月 日	年 月 日	年 月 日

NO.2012 2037712
