# Supplementary material for: Exposure to Sunlight Reduces the Risk of Myopia in Rhesus Monkeys
Source: PLoS One. 2015 Jun 1;10(6):e0127863. doi: 10.1371/journal.pone.0127863 (PMC4451516; doi:10.1371/journal.pone.0127863)
Supplement: S4 File — (PDF) [file pone.0127863.s004.pdf]

实验动物生产许可证

许可证号 SCXK(粤)2014-0010

单 位 名 称：广东蓝岛生物技术有限公司

法定代表人：韩日畴

设 施 地 址：广州市萝岗区九龙镇红卫广华路 33 号

适 用 范 围：普通级（食蟹猴、猕猴，9971 ㎡）

发证机关：广东省科学技术厅

二〇一四年六月十七日

有效期五年
