# Supplementary material for: Exposure to Sunlight Reduces the Risk of Myopia in Rhesus Monkeys
Source: PLoS One. 2015 Jun 1;10(6):e0127863. doi: 10.1371/journal.pone.0127863 (PMC4451516; doi:10.1371/journal.pone.0127863)
Supplement: S5 File — (PDF) [file pone.0127863.s005.pdf]

实验动物使用许可证

许可证号:SYXK(粤)2013-0088

单位名称：广东蓝岛生物技术有限公司

法定代表人：韩日畴

设施地址：广州市萝岗区九龙镇红卫广华路33号

适用范围：普通环境(猴、400㎡)

有效期五年

发证机关：广东省科学技术厅

二〇一三年[illegible]月二十一日
